# In Situ Reactive Extrusion of LDPE Films with Methacrylated Pyrogallol for Antimicrobial and Antioxidant Active Packaging

**DOI:** 10.3390/polym17030325

**Published:** 2025-01-25

**Authors:** Sharifa Salma Sulistiawan, Kambiz Sadeghi, Ritesh Kumar, Dowan Kim, Jongchul Seo

**Affiliations:** 1 Department of Packaging & Logistics, Yonsei University, 1 Yonseidae-gil, Wonju-si 26493, Republic of Korea; sharifasalma@yonsei.ac.kr; 2 School for Engineering of Matter, Transport, and Energy, Arizona State University, 11 501 E Tyler Mall, Tempe, AZ 85287, USA; kambiz_sadeghi@ymail.com; 3 Sri Guru Gobind Singh College of Commerce, Unversity of Delhi, Opposite TV Tower, Pitampura, Delhi 110034, India; riteshkumar@sggscc.ac.in; 4 Department of Food Processing and Distribution, College of Life Science, Gangneung-Wonju National University, 7 Jukheon-gil, Gangneung-si 25457, Republic of Korea; dowankim@gwnu.ac.kr

**Keywords:** reactive extrusion, industrial process, active packaging, antimicrobial, antioxidant

## Abstract

Reactive extrusion is a scalable technology for active packaging that promotes food quality and safety. This study investigated the grafting of a methacrylate pyrogallol (PGMC) active agent onto low-density polyethylene (LDPE) through an in situ reactive extrusion process with varying concentrations of PGMC (1, 3, and 5 wt.%). The addition of 5% PGMC increased the tensile strength of pure LDPE from 17.94 MPa to 22.04 MPa. The thermal stability of the samples remained unaffected after PGMC addition, and the grafting process did not interfere with the barrier properties of the LDPE films. Furthermore, 5% PGMC exhibited significant antimicrobial properties, showing 100% and 99.11% reductions in the microbial activity of Escherichia coli (Gram-negative) and Staphylococcus aureus (Gram-positive), respectively. Moreover, the LDPE film with 5% PGMC demonstrated the highest DPPH scavenging effect, reaching up to 85.71%. Therefore, LDPE-g-PGMC films (5%), with antimicrobial and antioxidant properties, have potential applications in non-migratory active packaging.

## 1. Introduction

Microbial and oxidative activities play significant roles in product deterioration, particularly in the food and pharmaceutical industries. These activities cause concerns, such as foodborne illnesses and waste problems. Various approaches, including chemical or physical preservation methods, regulating water activity, and maintaining specific atmospheric conditions, are often employed to extend the shelf lives of packaged goods. These measures are implemented to preserve the quality and safety of products and enhance their shelf lives [1,2,3,4]. Extending a product’s shelf life through packaging systems is preferred nowadays, especially with an increased consumer demand for “clean” labels. Regarding the consumer demand for reduced food additives in products, companies must adjust their strategies, focusing on modifying packaging [5].

Polyethylene, specifically low-density polyethylene (LDPE), is the most extensively used polymer for packaging. LDPE has diverse applications in flexible packaging, serving as an inner layer in multilayer packaging systems, as a food-safe contact material, and as a sealing layer in various packaging formats. The widespread application of LDPE as a packaging material is attributed to its flexibility, high transparency, moisture barrier properties, chemical resistance, and cost-effectiveness [6,7]. However, conventional LDPE lacks functional properties beyond these primary packaging properties. Therefore, numerous studies have been conducted to add active agents to LDPE polymers [8,9,10,11].

Active agents such as gallic acid [12], plant extracts [13], chitosan [14], and alumina [15] are often added to packaging materials to achieve functional properties such as oxygen scavenging, antimicrobial activity, ethylene scavenging, and moisture control [16]. Pyrogallol (PG) is a natural phenolic compound exhibiting antioxidant and antimicrobial properties owing to its hydroxyl functional groups [17]. However, introducing biomaterials such as PG into a polymer matrix might lead to undesired results such as non-uniform dispersion, the loss of end-product functionality, and a low processability for heat-induced processes. Ahn et al. enhanced the oxygen-scavenging ability of LDPE films by incorporating gallic acid using a compounding extruder [18]. They observed potent oxygen-scavenging properties; however, the mechanical properties of the films deteriorated as the gallic acid content increased. This outcome was caused by the agglomeration of the active agent, which is often caused by lack of compatibility and immiscibility. Furthermore, Promsorn and Harnkarnsujarit reported the compounding of unmodified PG into a polymer blend using twin-screw extrusion [19]. Films containing high concentrations of PG exhibited self-aggregation within the matrix, resulting in a non-homogeneity that decreased the mechanical properties and resulted in void spaces in the microstructures.

Blending active agents with a polymer matrix using conventional extrusion processes leads to undesirable properties such as poor interaction and agglomeration, resulting in poor mechanical properties, poor barrier properties, and migration issues [20,21,22,23]. Contrarily, functionalization reactions can be successfully performed using in situ reactive extrusion. This process enables strong chemical reactions in the polymer matrix, such as covalent bonding, cross-linking, and copolymerization, without requiring solvents [24,25,26]. Accordingly, the active agent is linked to the main-chain polymer through the reactive group, improving the compounding and overall properties of the finished product [27]. Reactive extrusion is a versatile alternative method for manufacturing active packaging because it is inexpensive, fast, and suitable for industrial production.

In our previous study, we developed a modified pyrogallol methacrylate (PGMC) with reactive functional groups. This modification aimed to improve the antimicrobial activity of PGMC while enhancing its thermal stability to endure the melt extrusion process, which usually performed at temperatures up to 200 °C [28]. Therefore, PGMC can be practically compounded with LDPE and is compatible with heat-induced industrial processes such as extrusion, injection molding, and polymerization. Specifically, the PGMC monomer retained the antimicrobial and antioxidant properties of PG, even after modification.

We have previously introduced non-migratory active packaging using photografting technology [29]. However, this technology is limited by its low-scale production and multistep processes, making it unfavorable for industrial purposes. Therefore, introducing non-migratory active packaging by adopting biomaterials using conventional fabrication processes ensures the integrity, scalability, functionality, and safety of active packaging. To our knowledge, no studies have incorporated modified PG into an LDPE matrix by in situ extrusion. This study introduces LDPE films grafted with PGMC monomers via in situ reactive extrusion using benzoyl peroxide (BPO) as an initiator. This study investigates the possibility of introducing modified bio-based additives into commonly used polymers using conventional fabrication methods. In this context, FTIR and ssNMR spectroscopy are used to analyze the non-migratory features of the resulting films, and the intense chemical reactions in their matrix are analyzed. The study also investigates the impact of varying the PGMC content on the chemical, physical, mechanical, and functional properties of the LDPE films. In addition, the antimicrobial and antioxidant properties of the films are assessed to provide insights into their potential applications as scalable non-migratory active packaging materials.

## 2. Materials and Methods

### 2.1. Materials

LDPE (pellets; grade 310S; MI 0.8 g/10 min) was obtained from Hanhwa Chemical Co., Ltd. (Seoul, Republic of Korea). PG, glycidyl methacrylate (GMA), triphenylphosphine (TPP), anhydrous ethyl alcohol, and butyl acetate were purchased from Daejung Chemicals (Siheung, Republic of Korea). p-Methoxyphenol (MEHQ) and BPO were purchased from Sigma-Aldrich (St. Louis, MO, USA). 2,2-diphenylpicrylhydrazyl (DPPH) was purchased from Alfa Aesar (Haverhill, MA, USA). Nutrient broth, MacConkey agar, tryptic soy broth, and tryptic soy agar were purchased from Duksan Pure Chemicals Co. Ltd. (Ansan, Republic of Korea). *Escherichia coli* DH5α (*E. coli*) was supplied by the Korean Culture Center of Microorganisms (Seoul, Republic of Korea), and *Staphylococcus aureus* ATCC 29,213 (*S. aureus*) was obtained from the American Type Culture Collection (Manassas, VA, USA).

### 2.2. Synthesis of PGMC Monomer

Pyrogallol (0.05 mol) was dissolved in a mixture of ethyl alcohol and butyl acetate (1:2, *v*/*v*), followed by the dissolution of triphenylphosphine (15 wt.%) and MEHQ (0.5%). Subsequently, GMA (0.05 mol) was added dropwise to the solution and purged with nitrogen for 20 min. Subsequently, the ring-opening reaction was conducted at (90 ± 5) °C in a nitrogen environment for 24 h. The PGMC monomers were precipitated using toluene and water to remove the unreacted components. The PGMC monomer was further purified by centrifugation at 4500 rpm for one hour. The dark brown viscous crude PGMC monomer was separated and stored in a refrigerator (4 °C to 6 °C).

### 2.3. Reactive Extrusion of LDPE-g-PGMC

**Figure 1:**
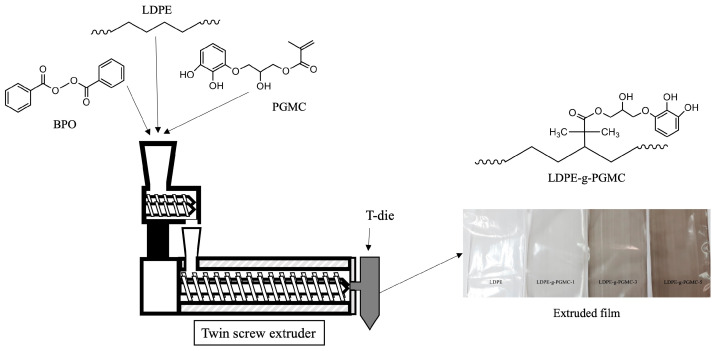
Grafting mechanism of PGMC into LDPE via reactive extrusion.

Figure 1 illustrates the grafting mechanism of PGMC and LDPE via reactive extrusion. Extrusion-grade LDPE was dried in a convection oven at 60 °C for 4 h. Subsequently, LDPE pellets were mechanically premixed with PGMC monomers (1, 3, and 5% *w*/*w*) and BPO (0.5% *w*/*w*) using a polyethylene bag. The exact composition of each sample is presented in Table 1. The mixture was then introduced into a laboratory-scale twin-screw extruder BA-19 (BauTech Co., Uijeongbu, Republic of Korea) at a length/diameter (L/D) ratio of 40:1 for homogeneous mixing and dispersion. The temperatures for processing were 90 °C for the header and 100, 100, 120, 120, 140, 140, and 140 °C for the seven subsequent heating zones. A T-die was attached to the end of the extrusion system to produce extruded LDPE-g-PGMC films. The obtained films had a thickness range from 0.07 to 0.09 mm. The extruded films were stored in polyethylene pouches under ambient conditions.

### 2.4. Characterization

Attenuated total reflection Fourier-transform infrared (ATR-FTIR) spectroscopy was employed to evaluate the chemical structures of the extruded films using a Spectrum 65 FT-IR (PerkinElmer Inc., Waltham, MA, USA) with a range from 4000 to 400 cm^−1^ in the 64-scan mode. Their chemical structures were evaluated using solid-state nuclear magnetic resonance (ssNMR) spectroscopy. ^13^C spectra were obtained using a UnityINOVA 600 MHz DS101 spectrometer (Varian Inc., Palo Alto, CA, USA). The X-ray diffraction (XRD) patterns of the samples were recorded on a high-resolution X-ray diffractometer SmartLab for film samples (Rigaku Co., Tokyo, Japan) using a CuKα source in the 2θ range from 2° to 60°. The crystallite size (Å) was calculated using the Scherrer Formula (1), as follows:

(1) $$D=\frac{K\lambda }{\beta cos\theta }$$

where K is the Scherrer constant (0.94), λ is the wavelength of the X-ray sources (0.154 nm), β is the full width at half maximum (FWHM, in radians), and θ is the diffraction angle of the peak.

The thermal stability of the film samples was evaluated by thermogravimetric analysis (TGA) using a TGA 4000 instrument (PerkinElmer Inc., Waltham, MA, USA). Furthermore, from 10 mg to 11 mg of the sample was heated from 30 °C to 800 °C at a rate of 10 °C/min under a nitrogen flow of 20 mL/min. The thermal properties of the films were analyzed using differential scanning calorimetry (DSC Q10, TA Instruments, New Castle, DE, USA). Enthalpy changes in the 5 mg sample were measured in the temperature range from 30 °C to 180 °C at a heating rate of 10 °C/min under a nitrogen atmosphere. The relative crystallinity from the DSC was calculated using Formula (2), as follows:

(2) $$X_{c}=\frac{{\Delta H}_{m}}{\Delta H_{100}}\times 100\%$$

where ΔH_m_ is the enthalpy absorbed by the sample during the heating process and ΔH_100_ is the enthalpy of 100% crystalline polyethylene (the value of 293 J/g) [30].

The densities of the extruded films were measured based on Archimedes’ principle using an Electronic Densimeter MD-300S (Alfa Mirage Co., Ltd., Osaka, Japan). The mechanical properties of the extruded films were measured using a universal testing machine (UTM QM100T-C, Qmesys Co., Uiwang-si, Republic of Korea) with a load cell of 20 kgf and a pulling rate of 200 mm/min. The oxygen transmission rate (OTR) was analyzed using the Oxygen Permeation Analyzer OxySense 8101 (Systech Illinois, Grand Chain, IL USA) at a temperature of 23 °C, 0% relative humidity, and the starting level of a 100 OTR value. Furthermore, the water vapor transmission rate (WVTR) was measured using the Water Vapor Permeation Analyzer AquaSense 7101 (Systech Illinois, Grand Chain, IL, USA) at a 90% relative humidity and a temperature of 37 °C.

The contact angles and surface free energies of the LDPE-g-PGMC films were measured using a Phoenix 300 contact angle goniometer (SEO Co., Ltd., Seoul, Republic of Korea) via the sessile drop method. The contact angles were measured at room temperature using deionized (DI) water and diiodomethane. Furthermore, the surface free energy was calculated using the instrument software through five different contact angle measurements for each sample.

The antimicrobial activities of the LDPE-g-PGMC samples were evaluated according to the JIS Z 2801:2010 standard [31]. *E. coli* and *S. aureus* were used as the Gram-negative and Gram-positive microorganisms, respectively. *E. coli* was grown on MacConkey agar, whereas *S. aureus* was grown on tryptic soy agar at 37 °C and 90% RH. A single colony of each bacterium was transferred to 10 mL of nutrient broth for *E. coli* and tryptic soy broth for *S. aureus*. The LDPE-g-PGMC films of each concentration were cut into 5 cm × 5 cm dimensions. The samples were sterilized using ethanol (70 wt.%), then UV irradiation for 15 min. Subsequently, 0.4 mL of the bacterial suspension was dropped onto the film surface and covered with a sterilized 4 cm × 4 cm pure polyethylene film. The stacked films were then incubated for 24 h at 37 °C and 90% RH. The samples were then sanitized with 0.9% saline solution, and serial dilutions were performed before each of the dilutions were cultured on agar plates. The number of colonies on each agar plate is reported as bacterial colony-forming units (CFUs). Finally, the percentage of reduction in microbial activity was calculated using Formula (3), as follows:

(3) $$Reduction\left(\%\right)=\frac{A-B}{A}\times 100$$

where A and B are the number of CFUs of viable microbial cells after 24 h for the control film (with 0% PGMC) and the LDPE-g-PGMC films, respectively.

The antioxidant activity of the LDPE-g-PGMC films was evaluated using the DPPH assay. DPPH (0.1 mM) was dissolved in ethyl alcohol in a light-free environment. Subsequently, the film samples were cut into 2 cm × 2 cm strips, submerged in a DPPH solution (4 mL), and incubated at room temperature in the dark. The absorbance of the solution at a wavelength of 517 nm was measured using UV–Vis spectrophotometry, and its progress was observed for up to 15 days.

### 2.5. Statistical Analysis

A wide range of tests were performed in this investigation, with at least three replications for every sample. The results presented in the tables are the mean values derived from these measurements, with each table also displaying the standard deviations. The equality of the means was assessed using equal-means hypothesis testing. Hypothesis tests were carried out via the ANOVA method, using the Tukey criteria for equal-variance assumptions, setting confidence intervals at 95% and significance levels at *p* < 0.05.

## 3. Results

### 3.1. Chemical Structure

**Figure 2:**
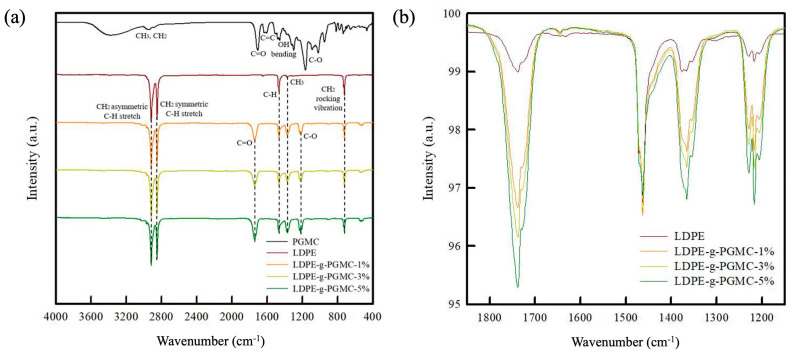
(**a**) FTIR spectra of LDPE, PGMC, and LDPE-g-PGMC films and (**b**) stacked FTIR spectra of LDPE-g-PGMC films.

The chemical structures of the LDPE-g-PGMC films were characterized using FT-IR by comparing them with pure LDPE and PGMC. Figure 2a illustrates the FTIR spectra of LDPE, PGMC, and the LDPE-g-PGMC films; their characteristic peaks are listed in Table 2.

All LDPE-g-PGMC films exhibited several characteristic peaks corresponding to LDPE chemistry, including asymmetric and symmetric C-H stretches at 2915 cm^−1^ and 2847 cm^−1^, a C-H bond at 1463 cm^−1^, CH_3_ umbrella bending at 1365 cm^−1^, and CH_2_ rocking vibration at 719 cm^−1^ [32]. The grafting of PGMC into the LDPE films was indicated by the characteristic peaks of PGMC, such as C=O stretching at 1738 cm^−1^ and a C-O bond at 1217 cm^−1^, which were slightly blue-shifted [33]. In addition, the FT-IR spectra showed an increase in the intensity of the C=O and C-O peaks in the LDPE-g-PGMC films with an increasing PGMC content (Figure 2b). The 1365 cm^−1^ peak, representing the CH_3_ of the LDPE backbone, also intensified with an increase in PGMC content, because it overlapped with the OH bending peak of the PGMC monomer [34]. Expectedly, the C=C bond of PGMC disappeared in all the LDPE-g-PGMC films, indicating that PGMC was successfully grafted onto LDPE through a free radical reaction [35,36].

**Table 2:** Chemical structure and wavelength corresponding to the FTIR peaks of LDPE, PGMC, and LDPE-g-PGMC films.

Sample	Peak (cm^−1^)	References
PGMC	3384 (OH stretching); 2932, 2886 (-CH_3_ & -CH_2_); 1702 (C=O); 1603, 944, 806 (C=C); 1477 (C-H); 1295 (C-C); 1164 (C-O-C); 1012 (C-O)	[33,37,38,39]
LDPE	2915 (asymmetric C-H); 2847 (symmetric C-H); 1463 (C-H); 1365 (-CH_3_ bending); 719 (CH_2_ rocking vibration)	[32,40,41]
LDPE-g-PGMC	2915 (asymmetric C-H); 2847(symmetric C-H); 1738 (C=O); 1463 (C-H); 1365 (-CH_3_ bending); 1217 (C-O); 719 (CH_2_ rocking vibration)	[34,42]

**Figure 3:**
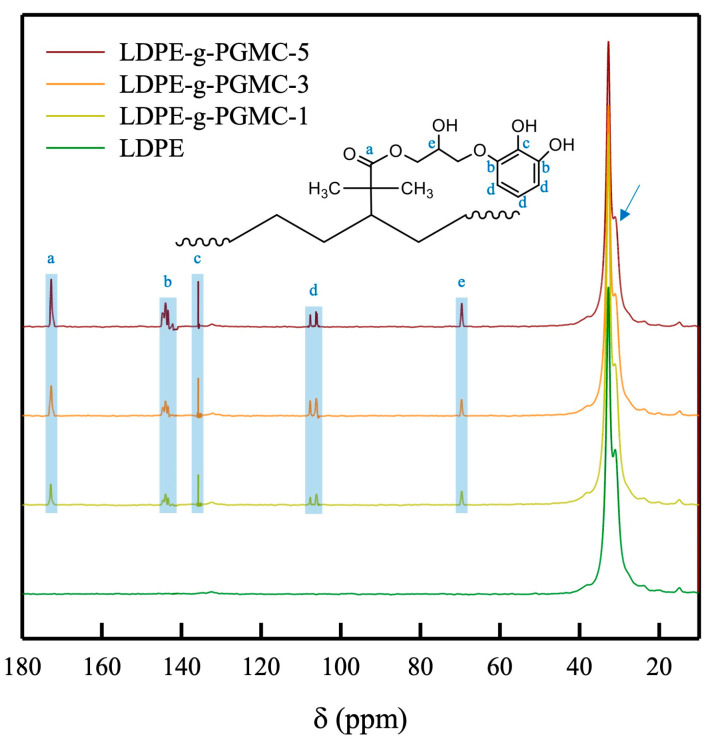
^13^C NMR spectra of LDPE-g-PGMC films with different PGMC concentrations.

Furthermore, the chemical structure was confirmed by carbon atom examination using ^13^C solid-state NMR, as depicted in Figure 3. The chemical shifts at δ = 33 ppm and δ = 31 ppm correspond to orthorhombic crystalline and amorphous regions of LDPE, respectively [43,44,45,46]. The chemical shifts at δ = 173 ppm and δ = 69 ppm confirm the presence of C=O [47,48,49,50,51] and C-OH structures [48,52,53] in the methacrylate group of PGMC. In addition, the chemical shifts at δ = 144, 135, 108, and 106 ppm confirm the carbon bonds from the phenolic structure of the PG of the PGMC, labeled using “b”, “c”, and “d”, respectively [48,53,54,55]. All LDPE-g-PGMC films showed identical chemical shifts, and their intensities increased with an increasing PGMC concentration. However, the chemical shift at δ = 31 ppm decreased with the addition of PGMC, indicating a decrease in the amorphous region of LDPE (labeled by an arrow) [54]. This outcome indicates that the grafting of PGMC onto LDPE occurred only in the amorphous regions of the polymer [56,57]. FTIR and NMR analyses indicated that PGMC with C=C bonds was successfully grafted onto LDPE via in situ melt extrusion. It is worth noting that there were no peaks corresponding to appendages or any unwanted by-products in the FTIR and NMR spectra, indicating that the materials in the feed were compatible with the in situ reactive extrusion.

In situ reactive extrusion is a versatile method for incorporating functional compounds into polymers via covalent linkage formation. In our study, the BPO initiator played a crucial role in inducing chemical reactions between the LDPE backbone and the PGMC. When heat was applied during extrusion, the BPO initiator underwent cleavage, thus forming radical species. These radical species then interacted with the LDPE backbone by abstracting hydrogen atoms, thus creating radical LDPE. The presence of radical LDPE reacted with the acrylic group present in the PGMC monomer, as supported by the disappearance of the C=O bond of PGMC in the LDPE-g-PGMC films. This reaction occurred via a radical addition mechanism, in which the radical LDPE reacted with the acrylic group, thus forming a covalent linkage between LDPE and PGMC. The likelihood of this reaction between the acrylic group and LDPE might be attributed to their nonpolar compatibility, indicating that their nonpolar nature facilitates the reaction and formation of covalent linkages [58,59].

**Figure 4:**
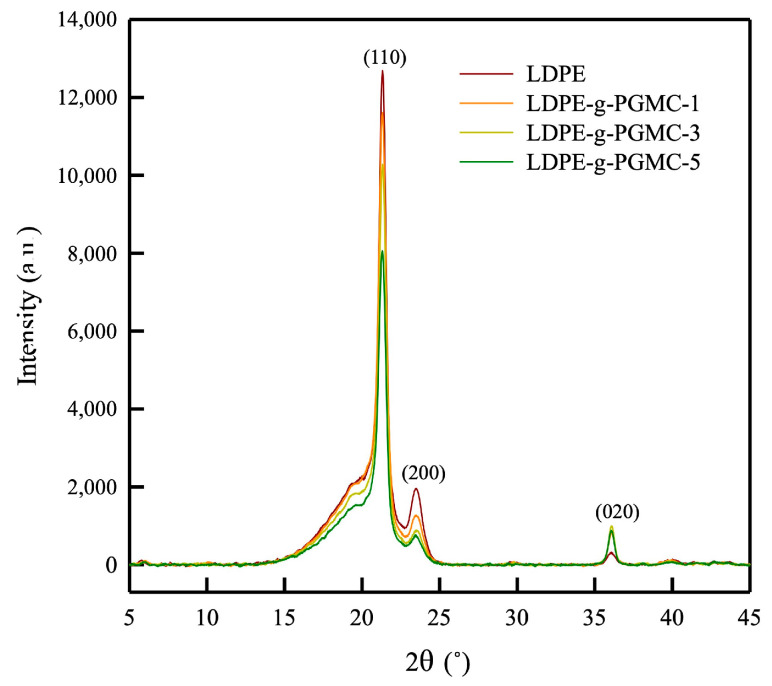
X-ray diffraction spectra of LDPE and LDPE-g-PGMC films.

**Figure 5:**
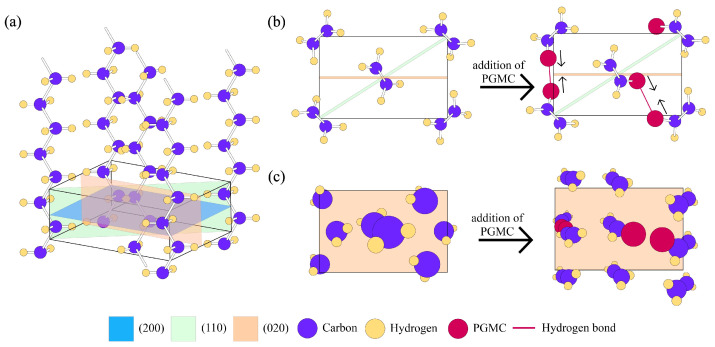
(**a**) Orthorhombic crystal structure of LDPE; (**b**) projection of unit cell down *z*-axis of LDPE (left) and LDPE-g-PGMC (right); and (**c**) projection of unit cell on the (020) plane of LDPE (left) and LDPE-g-PGMC (right).

The XRD results for all the samples are shown in Figure 4, and the crystal structure parameters are listed in Table 3. The crystalline structure of the polymer is illustrated in Figure 4. The diffractogram of LDPE exhibited characteristic peaks at 2θ of 21.3°, 23.4°, and 36.4°, corresponding to the diffraction planes of orthorhombic crystals at (110), (200), and (020) in polyethylene, respectively (Figure 5a) [60,61,62]. In addition, a broad shoulder at 2θ of about 19.9° can also be observed, indicating the semi-crystalline nature of LDPE [63]. Consistent peak positions were observed in all LDPE-g-PGMC samples, indicating that the addition and grafting of PGMC did not alter the basic crystal structure of LDPE [64]. The intensities of the peaks from the (110) plane at 21.3° and the (200) plane at 23.4° decreased as the PGMC content increased. However, as the PGMC content increased, the peak intensity of the (020) plane at 36.4° increased. This outcome indicates a change in the orientation of the orthorhombic crystals in the LDPE-g-PGMC films [65]. Table 3 shows that the crystallite size in the (020) lattice plane increased with an increasing PGMC content. Grafting PGMC onto LDPE lowered the ordering in the crystals in the (110) and (200) planes, although there was a tendency towards more ordered chains in the (020) plane of the polymer (Figure 4). Intermolecular interactions between hydrogen bonds and the aromatic ring of PGMC in the side chain of the polymer induced the ordering of the lamellar structure of the polymer in that direction [66].

### 3.2. Thermal Properties

**Figure 6:**
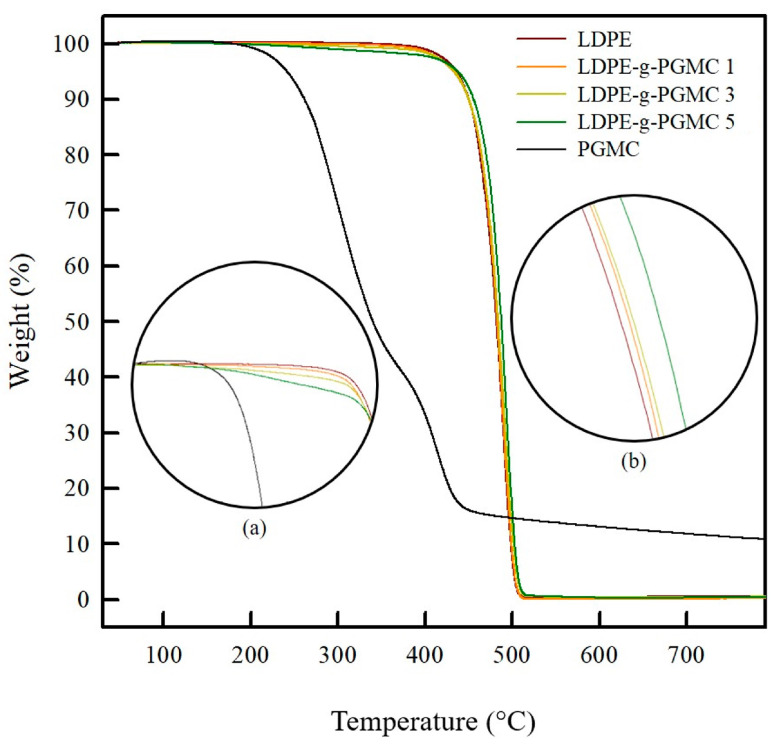
TGA analysis of pure LDPE, PGMC, and LDPE-g-PGMC films: (**a**) zoom of selected area around 200–400 °C and (**b**) zoom of selected area around 400–500 °C.

As shown in Figure 6, the thermal stability of the LDPE-g-PGMC films was investigated using TGA. PGMC showed a two-step thermal degradation process, as follows: the release of CO_2_ from the degradation of the hydroxyl group at around 200 °C and the degradation of the ester group at approximately 350 °C [67,68,69,70]. However, LDPE showed one-step steep thermal degradation with an onset temperature of 400 °C, likely corresponding to the chain scission of polyethylene [71,72,73]. It is worth noting that all LDPE-g-PGMC films exhibited a decomposition pattern similar to that of LDPE, indicating that the introduction of PGMC did not change their thermal degradation behavior. This aspect supports the successful grafting and string addition of PGMC to LDPE via covalent bonding [74,75,76]. However, all of the LDPE-g-PGMC films exhibited a slight weight loss within the temperature range of 230–450 °C, which became more noticeable with increasing concentrations of PGMC (highlighted in a circle in Figure 6. This outcome may be related to the release of CO_2_ by the decomposition of the PGMC [67,68,77].

**Figure 7:**
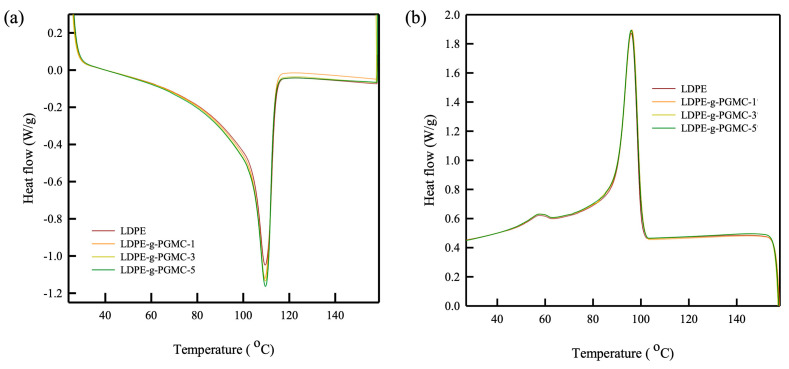
DSC analysis of pure LDPE, PGMC, and LDPE-g-PGMC films: (**a**) heating process and (**b**) cooling process.

The thermal properties of the LDPE-g-PGMC films were further examined using DSC, as shown in Figure 7 and Table 4. LDPE exhibited a single peak at a melting temperature of approximately 109 °C and a single peak at a recrystallization temperature of 96 °C. Regardless of the introduction of PGMC, the thermograms of the LDPE-g-PGMC films exhibited a pattern similar to that of LDPE. However, with an increase in the PGMC content, both the melting enthalpy and recrystallization enthalpy of the LDPE-g-PGMC films exhibited an upward trend, rising from 97.1 J/g to 105.9 J/g and from 68 J/g to 70 J/g, respectively. A higher enthalpy indicates a higher crystallinity or increased chain ordering in the polymer matrix, which agrees with the greater ordering of crystallites in the (020) plane from the XRD analysis owing to the presence of hydrogen bonding and an aromatic ring [78,79]. Notably, the addition of PGMC did not affect the LDPE film processability because its melting behavior was maintained. Additionally, no further peaks corresponding to degradation or undesirable reactions were observed, indicating that the materials were thermally stable during heat induction processes such as extrusion.

### 3.3. Film Density

The film density increased from approximately 0.857 g/cm^3^ for pure LDPE to 0.986 g/cm^3^ for LDPE-g-PGMC-5%, as presented in Table 5. The film density increased with an increasing PGMC content. This higher film density may have been related to heavier elements, such as oxygen, added to the LDPE-g-PGMC films. Furthermore, combined with the XRD analysis, the close packing in the structure caused by intermolecular interactions may have increased the density of the LDPE-g-PGMC films [80].

### 3.4. Mechanical Properties

**Figure 8:**
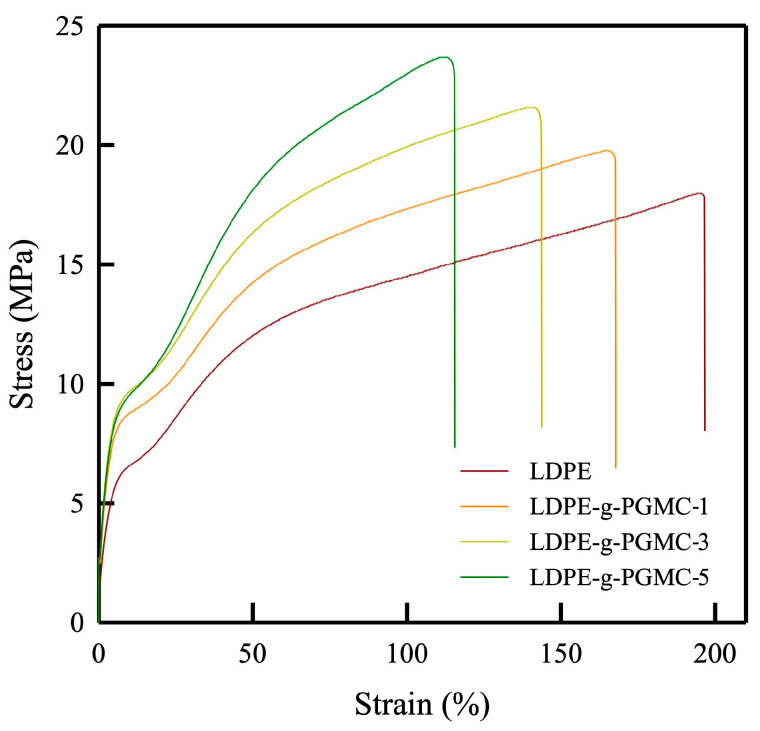
Stress–strain curve of pure LDPE and LDPE-g-PGMC films.

The mechanical properties of the LDPE-g-PGMC films are presented in Table 5 and Figure 8. The pure LDPE showed a typical ductile profile among the stress–strain curves, in which LDPE exhibited the highest elongation at break, lowest tensile strength, and lowest Young’s modulus among the samples. The LDPE-g-PGMC films lost ductility after the grafting process, resulting in a more brittle profile than the pure LDPE films. The tensile strength was increased from 114.3 MPa for the pure LDPE to 179.1 MPa for LDPE-g-PGMC-5%. Meanwhile, the elongation at break of the LDPE-g-PGMC films decreased from 188.2% for the pure LDPE to 115.5% for LDPE-g-PGMC-5%. The change in the mechanical properties demonstrated a clear dependence on the composition, with higher concentrations of PGMC leading to an increased tensile strength and decreased elongation at break. Specifically, as the concentration of PGMC increased, the LDPE-g-PGMC films exhibited a higher resistance to deformation under tension but a reduced ability to elongate before breaking. Generally, adding bulky groups such as aromatic rings to the polymer matrix increases the rigidity of polymers [81]. Therefore, bulky benzene from the PGMC may have also contributed to these property changes. Furthermore, intermolecular hydrogen interactions limit the mobility of the polymer chain, resulting in a higher stiffness and overall tensile strength of films [82].

### 3.5. Contact Angle

**Figure 9:**
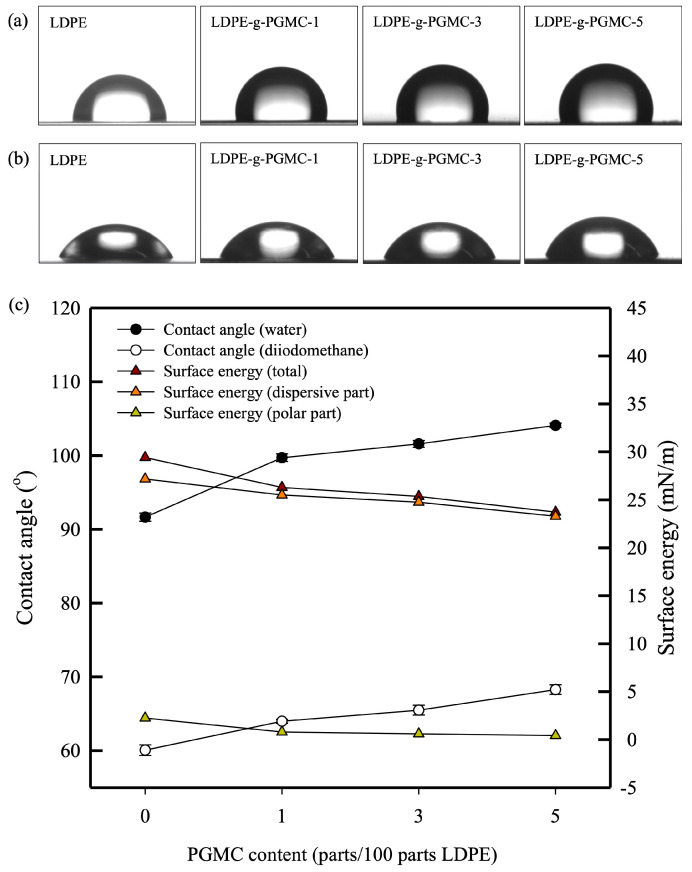
(**a**) Contact angle of film samples with 0, 1, 3, and 5% content of PGMC (water), (**b**) contact angle of film samples with 0, 1, 3, and 5% content of PGMC (diiodomethane), and (**c**) contact angle and total surface energy of the film samples.

The surface energy and contact angles of LDPE-g-PGMC are presented in Figure 9. DI water and diiodomethane were used as polar and dispersive components, respectively. The pure LDPE film exhibited the lowest contact angles of 91.7° for water and 60.1° for diiodomethane. These values increased to 104.1° and 68.3° for water and diiodomethane, respectively, in the LDPE-g-PGMC films. According to the change in the contact angle value, the total surface energy decreased from 29.4 mN/m to 23.7 mN/m with the addition of PGMC. The reduction in surface energy was due to polar and dispersive component reduction. The surface energy of the polar portion, which was significantly affected by chemical affinity, might have been decreased owing to the presence of an ester group in PGMC, which imparted a higher hydrophobicity to the films [83,84], whereas the surface energy of the dispersive portion was affected by the surface roughness of the film samples [85].

### 3.6. Barrier Properties

The oxygen and water permeabilities of the LDPE-g-PGMC films are listed in Table 5. The OTR values in film samples were decreased from 2237 cc/m^2^·day to 1658 cc/m^2^·day with the PGMC addition. The WVTR values decreased from 1.8 g/m^2^·day for the pure LDPE to 1.6 g/m^2^·day for LDPE-g-PGMC-5%. Gas permeation into the polymeric matrix occurred within the amorphous phase [86]. The presence of the OH group and the aromatic ring in the PGMC structure contributed to a more compact structure in the (020) plane (Figure 3), which reduced the free volume and blocked diffusion pathways [68,87]. Therefore, the increase in the oxygen and water vapor barrier properties may have been caused by the lower free volume available in the LDPE-g-PGMC films with a higher PGMC content as the amount of hydrogen and aromatic rings increased. Furthermore, the reduction in the polar and dispersive surface energies in the LDPE-g-PGMC films (Figure 8) might have lowered the permeability of the film surface [88].

### 3.7. Antimicrobial Properties

The antimicrobial properties of the LDPE-g-PGMC films against *E. coli* (Gram-negative) and *S. aureus* (Gram-positive) are presented in Table 6. The pure LDPE film without PGMC exhibited the highest number of microbes, indicating that the control sample did not exhibit antimicrobial activity. As PGMC was added to LDPE, the microbial number decreased significantly. LDPE-g-PGMC films containing 5% PGMC reduced their microbial activity by up to 99.11% and 100% against *S. aureus* and *E. coli*, respectively.

This potent antimicrobial property is due to the presence of PG in PGMC. The phenolic compounds generated hydroxyl radicals owing to alkyl substitution caused the function of the bacterial cell to be destabilized [89]. Furthermore, the antimicrobial activity of polyphenols, such as PG, was discovered to target the bacterial cell membrane and adsorb onto the surface of the bacterial cell wall [90].

### 3.8. Antioxidant Capacity

**Figure 10:**
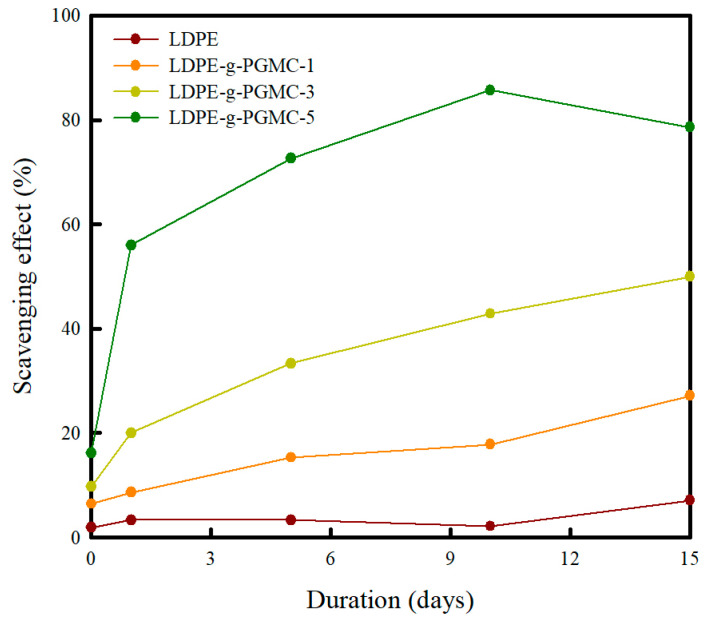
DPPH scavenging effect (%) of LDPE-g-PGMC films with different PGMC contents.

The free radical scavenging activity of the LDPE-g-PGMC films was evaluated using the DPPH assay for 15 days. The changes in the percentage of the scavenging effect were recorded and are presented in Figure 10. The pure LDPE film exhibited an insignificant change compared to the DPPH solution throughout the storage period, indicating that it did not have an antioxidant capability. In contrast, the absorbance of the LDPE-g-PGMC film decreased throughout the storage period. The LDPE-g-PGMC-1% and LDPE-g-PGMC-3% samples displayed the highest scavenging effects after 15 days, reaching 27% and 50%, respectively. Furthermore, LDPE-g-PGMC-5% exhibited a higher scavenging effect of 56% after only one day of storage. It reached its highest scavenging effect of approximately 85.71% on day 10, reached equilibrium, and was maintained at 78.5% on day 15.

Polyphenols, such as PG, exhibit antioxidant activity by donating hydrogen and atoms from hydroxyl groups to react with radicals [91,92]. The LDPE-g-PGMC films with higher PGMC concentrations exhibited more potent antioxidant properties because they contained more hydroxyl groups within their structure [93]. A slow release of antioxidant activity is preferred in some systems, such as fatty foods with a relatively long shelf life, because this can promote more effective antioxidant properties [94,95]. The LDPE-g-PGMC films were able to maintain their antioxidant activity for 15 days, which indicates that they could delay the oxidation of products for a relatively long storage time, providing better antioxidant activities.

## 4. Conclusions

This study successfully demonstrated the feasibility of incorporating biomaterials such as PG into LDPE using a scalable conventional method known as in situ reactive extrusion. The incorporation of modified biomaterials into the polymers resulted in active films with well-dispersed biomaterials and significant functional properties according to a chemical assessment of the films. The LDPE-g-PGMC films exhibited strong interactions within their structures, leading to a more ordered and compact arrangement. This enhanced structural organization improved their thermal stability, mechanical properties, and film barrier properties.

Notably, the LDPE-g-PGMC film with the highest PGMC content exhibited the highest reduction in microbial activity against *E. coli* and *S. aureus*. Furthermore, the LDPE-g-PGMC films exhibited a strong scavenging effect throughout the 15 days of storage. In conclusion, these LDPE-g-PGMC films demonstrate a good potential for use in antimicrobial and antioxidant food packaging applications. The incorporation of PGMC into LDPE via reactive extrusion offers a promising approach for developing functional packaging materials that can enhance food safety and extend shelf life through a non-migratory principle.

## Figures and Tables

**Table 1:** Sample name and compositions of the LDPE-g-PGMC films prepared in this study.

Name	LDPE Resin (g)	Benzoyl Peroxide (g)	PGMC (g)	Film Thickness (mm)
LDPE	300	0	0	0.075 ± 0.001
LDPE-g-PGMC-1	300	1.5	3	0.083 ± 0.001
LDPE-g-PGMC-3	300	1.5	9	0.077 ± 0.002
LDPE-g-PGMC-5	300	1.5	15	0.081 ± 0.005

**Table 3:** Crystal structure parameters of LDPE and LDPE-g-PGMC samples.

Samples	Lattice Plane	2θ (°)	FWHM (°)	Crystallite Size (Å)	D-Spacing (Å)
LDPE	110	21.3	0.536	1.5	4.2
	020	36.4	0.636	1.3	2.5
LDPE-g-PGMC-1	110	21.3	0.574	1.4	4.2
	020	36.4	0.516	1.6	2.5
LDPE-g-PGMC-3	110	21.3	0.546	1.5	4.2
	020	36.4	0.484	1.7	2.5
LDPE-g-PGMC-5	110	21.3	0.554	1.5	4.2
	020	36.4	0.498	1.7	2.5

**Table 4:** Thermal properties comparison of LDPE and LDPE-g-PGMC samples.

Samples	T_m_ (°C)	∆H_m_ (J/g)	T_c_ (°C)	∆H_c_ (J/g)	Xc (%)
LDPE	109.4 ± 0.1	97.1 ± 1.8	96.2 ± 0.1	68.0 ± 1.0	33.1
LDPE-g-PGMC-1	109.4 ± 0.2	104.9 ± 2.8	96.2 ± 0.1	70.1 ± 3.8	35.8
LDPE-g-PGMC-3	109.4 ± 0.1	105.8 ± 0.9	96.2 ± 0.1	71.1 ± 2.2	36.1
LDPE-g-PGMC-5	109.6 ± 0.2	105.9 ± 1.4	96.1 ± 0.1	70.0 ± 1.4	36.2

**Table 5:** Physical properties of pure LDPE and LDPE-g-PGMC films.

Samples	Density (g/cm^3^)	E ^1^ (MPa)	TS ^2^ (MPa)	EB ^3^ (%)	OTR (cc/m^2^·Day)	WVTR
						(g/m^2^·Day)
LDPE	0.857 ± 0.03 ^b^	114.3 ± 5.9 ^b^	17.9 ± 0.6 ^c^	188.2 ± 6.1 ^a^	2337 ± 20.4 ^a^	1.8 ± 0.01 ^a^
LDPE-g-PGMC-1	0.941 ± 0.03 ^a^	157.6 ± 6.4 ^a^	19.5 ± 0.4 ^b^	163.2 ± 3.4 ^b^	2030 ± 18.5 ^b^	1.7 ± 0.02 ^b^
LDPE-g-PGMC-3	0.962 ± 0.03 ^a^	169.9 ± 7.1 ^a^	19.9 ± 1.2 ^b^	135.4 ± 6.1 ^c^	1883 ± 44.9 ^c^	1.7 ± 0.01 ^b^
LDPE-g-PGMC-5	0.986 ± 0.03 ^a^	179.1 ± 7.8 ^a^	22.1 ± 1.1 a	115.5 ± 0.7 ^d^	1658 ± 33.0 ^d^	1.6 ± 0.01 ^c^

Values in the same column with different lowercase letters are significantly different (*p* < 0.05). The same as follows. ^1^ Young’s modulus, ^2^ tensile strength, and ^3^ elongation at break.

**Table 6:** Antimicrobial activities of LDPE and LDPE-g-PGMC films.

		LDPE	LDPE-g-PGMC-1	LDPE-g-PGMC-3	LDPE-g-PGMC-5
*E. coli*	Digital image	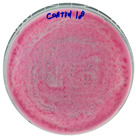	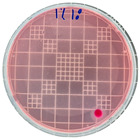	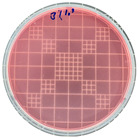	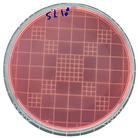
	Concentration (CFU/mL)	2.05 × 10^5^	2.00 × 10	0	0
	Reduction (%)	–	99.99	100	100
*S. aureus*	Digital image	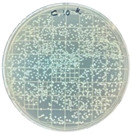	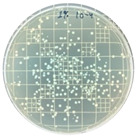	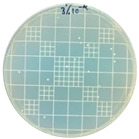	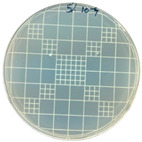
	Concentration (CFU/mL)	2.25 × 10^8^	2.40 × 10^7^	7.00 × 10^6^	2.00 × 10^7^
	Reduction (%)	–	89.33	96.86	99.11

## Data Availability

The original contributions presented in this study are included in the article. Further inquiries can be directed to the corresponding author.
